# Comparison of Surface Strains of Polymeric Frameworks for Fixed Implant-Supported Prostheses: A Digital Image Correlation Study

**DOI:** 10.3390/ma18081700

**Published:** 2025-04-09

**Authors:** Ana Messias, Maria Augusta Neto, Ana Paula Piedade, Ana Amaro, Jack T. Krauser, Fernando Guerra

**Affiliations:** 1 Centre for Mechanical Engineering, Materials and Processes (CEMMPRE), Department of Dentistry, Faculty of Medicine, University of Coimbra, 3000-075 Coimbra, Portugal; 2 Centre for Mechanical Engineering, Materials and Processes (CEMMPRE), Department of Mechanical Engineering, Faculty of Sciences and Technology, University of Coimbra, 3030-788 Coimbra, Portugal; augusta.neto@dem.uc.pt (M.A.N.); ana.piedade@dem.uc.pt (A.P.P.); ana.amaro@dem.uc.pt (A.A.); 3 Implant Center of the Palm Beaches, West Palm Beach, FL 33408, USA; jtkrauser@gmail.com; 4 Centre for Innovation and Research in Oral Sciences (CIROS), Department of Dentistry, Faculty of Medicine, University of Coimbra, 3000-075 Coimbra, Portugal; fguerra@ci.uc.pt

**Keywords:** implant-supported fixed prosthesis, polymeric frameworks, PEEK, PEKK, PMMA, fibre-reinforced composite, full-arch rehabilitations

## Abstract

The gold standard materials used for frameworks of full-arch implant-supported fixed prostheses (ISFPs) have traditionally been metal alloys, but recently, high-performance polymers such as polyetherketones and fibre-reinforced resins have been gaining popularity despite the lack of evidence of load-bearing capacity. The aim of the present study was to evaluate the displacements and strains of milled polymeric frameworks for full-arch ISFPs using 3D digital image correlation. Methods: Twelve frameworks were milled from four polymeric materials (three per group): polyetheretherketone (PEEK), polyetherketoneketone (PEKK), poly(methyl methacrylate) (PMMA) and fibre-reinforced composite (FRC). Each framework was fitted with titanium links and screwed to implant analogues embedded in resin and tested for static load-bearing capacity up to 200N. Displacements were captured with two high-speed photographic cameras and analysed with a video correlation system on three spatial axes, U, V, and W, along with principal tensile, compressive and von Mises strains. Results: PEEK exhibited the highest displacement, indicating greater flexibility, while FRC showed the lowest displacement, suggesting enhanced rigidity. Von Mises strain analysis revealed that PMMA and PEEK experienced higher strain, whereas PEKK and FRC demonstrated lower strain distribution. Bayesian ANOVA provided strong evidence for material differences. Conclusion: FRC exhibited superior load-bearing characteristics, reinforcing its potential as a viable clinical alternative to metal-based ISFPs.

## 1. Introduction

Edentulism, defined as the absence of natural teeth in the oral cavity, has a huge impact on the physical, social and psychological dimensions of an individual [1,2,3]. It is considered by the World Health Organization a source of impairment and disability and has been declared as one of the public health burdens of the 21st century. Even though the understanding of the epidemiology of tooth loss is still incomplete, a recent analysis of the Global Burden of Diseases Study estimates that 0.35 billion people are affected by edentulism, representing more than 4.5% of the world population [4,5].

The prevalence of edentulism has led to an increased demand for prosthetic treatments addressing the issues caused by missing teeth, which can vary depending on an individual’s perception of function, aesthetics or psychological well-being.

Prosthetic treatment options for complete edentulous patients range from conventional removable dentures to implant-retained overdentures and implant-supported fixed prostheses (ISFPs). Usually, ISFPs are supported on four to six implants and present a framework that is screwed to the implant or underlying meso-abutment. The framework is designed to support and distribute the stress equally between the implants and must possess adequate mechanical strength, dimensional stability and resistance to wear while also allowing for aesthetic integration with veneering materials such as ceramic or polymers (acrylic resin) to adequately simulate teeth and gingiva.

The gold standard materials used for frameworks of full-arch ISFPs have traditionally been metal alloys, such as cobalt-chromium or titanium alloys, due to their high mechanical strength and fatigue resistance. These metal alloys have good clinical performance but present some disadvantages mainly related to the unesthetic appearance of metal and the risk of failure at the framework–veneering interface [6,7,8,9]. In addition, concerns have been raised regarding transition metal allergies associated with these alloys, particularly in patients with sensitivities to nickel or chromium, which can lead to inflammatory responses and complications [10,11].

The limitations associated with metallic frameworks have driven the search for metal-free alternatives, such as all-ceramic, high-performance polymers and fibre-reinforced composites (FRCs), which offer enhanced aesthetics, favourable biomechanical properties and improved biocompatibility.

Among the polymers that have been explored for ISFP frameworks, high-performance polymeric materials such as polyetheretherketone (PEEK) and polyetherketoneketone (PEKK) have gained significant attention due to their low density, chemical stability and shock-absorbing properties [12,13,14,15]. Notably, their modulus of elasticity (PEEK: ~4.4 GPa, PEKK: ~3.4 GPa) is closer to that of bone than traditional metal alloys (~110–120 GPa for Co-Cr, ~100 GPa for titanium), potentially reducing stress shielding effects and promoting more physiological load transfer to the implants [16,17,18,19,20,21].

FRCs, consisting of a polymer matrix reinforced with glass or carbon fibers, have demonstrated superior mechanical performance, including higher flexural strength (~26 GPa) and improved resistance to fracture and fatigue degradation [22]. These properties make FRCs attractive for prosthetic frameworks, as they provide a balance between strength and flexibility, reducing the risk of framework fractures while allowing some shock absorption under occlusal loads. Additionally, compared to PEEK and PEKK, FRCs exhibit a higher load-bearing capacity, making them particularly promising for full-arch rehabilitations in functionally demanding patients [20,23,24,25,26,27,28].

However, despite their promising attributes, the mechanical performance of polymer-based ISFP frameworks remains a topic of debate. Previous studies have reported concerns regarding their long-term stability, resistance to deformation and overall structural integrity under functional loading conditions. On one hand, differences in displacement and strain behaviour between polymeric materials may increase the susceptibility to stress- and fatigue-induced cracking over time, thus influencing clinical outcomes, prosthesis longevity and patient satisfaction [17,27,29,30,31]. On the other hand, the low surface hardness of polymers used in dentistry leads to wear and surface scratching of structures after short periods of function, increasing surface roughness. This not only compromises aesthetics but also facilitates bacterial adhesion and accumulation and poses hygienic challenges [32,33,34,35,36,37,38,39,40].

To date, limited data are available comparing the biomechanical behaviour of different polymeric framework materials under static loading conditions. Understanding their mechanical performance is crucial for optimising material selection and improving the clinical predictability of metal-free ISFPs. Therefore, this study aims to evaluate and compare the displacement and strain distribution of polymeric frameworks for full-arch mandibular ISFPs under static loading. By analysing the mechanical response of these materials, we seek to provide insights into their potential clinical implications and guide future developments in prosthetic rehabilitation.

## 2. Materials and Methods

A total of 12 frameworks for full-arch maxillary ISFPs supported on 5 implants were milled in four different polymeric materials: polyetheretherketone (G1-PEEK), polyetherketoneketone (G2-PEKK), poly(methyl metacrilate) (G3-PMMA) and fibre-reinforced composite (G4-FRC).

**Figure 1:**
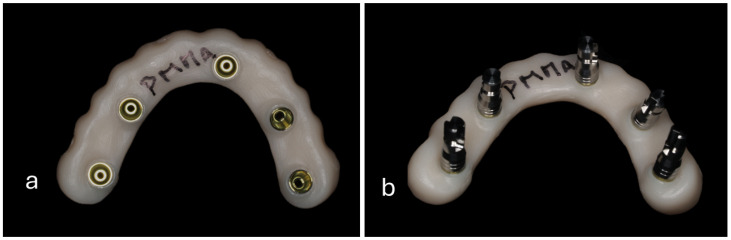
Cervical view of a G3-PMMA sample with (**a**) titanium copings for multi-unit abutments and (**b**) multi-unit implant analogues screwed to the framework.

Five titanium links for multi-unit abutments (Link Tibase Cinta 1.90 Mm Plat. Ø4.80—Eff6811.01.1, EFF Dental Components, São Paulo—Brazil) were cold-welded to each framework and then screwed at 35 N/cm^2^ to multi-unit implant analogues (Plat. Ø4.80—Eff311.01, EFF Dental Components, São Paulo—Brazil), as exemplified for a sample of G3-PMMA in Figure 1a,b.

All frameworks were air-sprayed with airbrush pro-colour ink (Hansa Airbrush, Norderstedt, Germany) to produce a non-repetitive, isotropic speckle pattern with high contrast for full-field measurement of displacements and deformation analysis with 3D digital image correlation.

**Figure 2:**
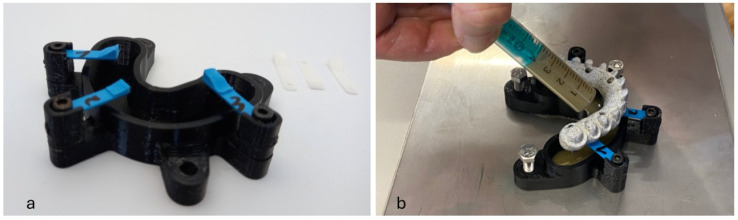
(**a**) Horseshoe container with supports (blue) for (**b**) embedding the implant analogues in acrylic resin ensuring parallelism of the occlusal plane.

Implant analogues were then embedded in Technovit^®^ 4000 (Heraeus Kulzer, Wehrheim, Germany), a modified fast-curing polyester-based resin with inorganic fillers for improved hardness, low shrinkage and excellent adhesion to metal surfaces, using a horseshoe-shaped container with supports to ensure parallelism to the horizontal plane of the occlusal surface of all frameworks, as shown in Figure 2a,b, while guaranteeing a straight central position and testing geometry with 3 mm distance between implant shoulder and the first implant-analogue–resin contact, in accordance with ISO 14801 [41].

**Figure 3:**
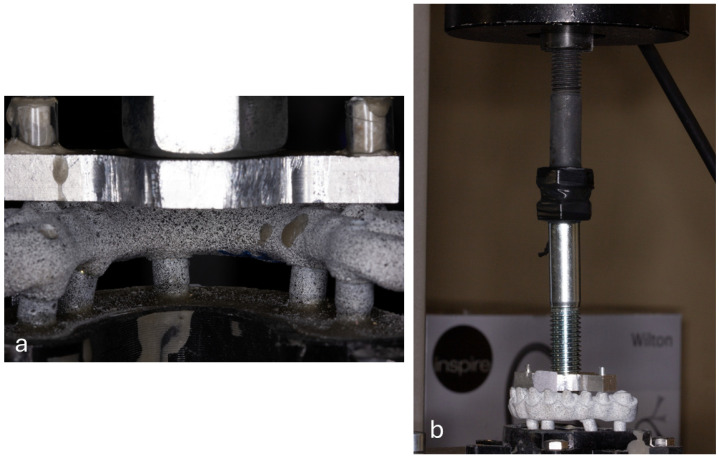
(**a**) Lingual view of the metallic plate used to apply uniform load over the framework and (**b**) general view of the experimental setup with the knee-joint connection between the load cell and metallic plate to ensure that the applied load was perpendicular to the occlusal plane of the frameworks.

Each specimen was fitted into a fixed metallic fixture and aligned to the load application centre of a universal test machine (AG-I Shimadzu^®^; Shimadzu Scientific Instruments, Columbia, MD, USA). The frameworks were tested for static load-bearing capacity using a metallic plate for homogenous load distribution across the framework, as represented in Figure 3a, and a knee-joint connection between the load cell and the metallic plate to ensure that the applied load was perpendicular to the occlusal plane of the frameworks (Figure 3b).

The load was applied increasingly at 0.5 mm/min velocity until the maximum force of 200 N was reached, as found in previous studies on implant-supported full-arch rehabilitations [42,43,44]. To isolate the possible effects of screw untightening on the implant–abutment stability and to prevent a possible bias derived from mechanical fatigue, no repetitive load was applied.

Photographs of the speckle patterns under the progressive loads were captured with two high-speed photographic cameras (Stingray F-504B ASG, Allied Vision, Andover, MA, USA) at the maximum resolution of 1624 × 1224 pixels (4.4 mm pixel size) and a maximum frame rate of 19 frames per second. The two cameras were positioned symmetrically about the framework with the stereo angle below 45° to keep magnification constant and to provide synchronised stereo images of the assemblies.

Calibration of the stereo system, which reflects the capacity of the transformation algorithm to convert deformation into displacements, was performed using a 30–45 mm field of view target with a 12 × 9 dot grid and 4 mm spacing. All tests presented final calibration scores lower than 0.1 pixels, indicating that the average error (in pixels) between the position where a target point was found in the image and the theoretical position where the mathematical calibration model placed the point was within the reasonable threshold and therefore with no systematic errors.

Captured speckle images were then analysed with the video correlation system Vic-3D 2012 (Correlated Solutions™, Columbia, SC, USA). Removal of rigid-body displacement was made considering the three-point displacement transformation by keeping three points of the surface of the implant stationary, thus reflecting framework displacements in relation to those points in the transformed U (lateral), V (vertical) and W (anterior–posterior) axes. For each specimen, the magnitude of displacement (or resultant displacement) was determined using the relation $Magnitude=\sqrt[{2}]{U^{2}+V^{2}+W^{2}}$ considering each point of the surface.

Mean values of the magnitude of displacement, principal tensile (Ɛ1) and compressive (Ɛ2) strains and von Mises strains (ƐVM) of the speckle patterns were collected at 50 N, 100 N, 150 N and 200 N.

Statistical analyses were performed with RStudio “Kousa Dogwood” Release (cf37a3e5, 2024-12-11) for Windows using a Bayesian approach to account for the small sample size and quantify uncertainty in parameter estimates. A Bayesian ANOVA was performed to assess differences between groups at 200 N in the magnitude of displacement and von Mises strains. The analysis was implemented using *BayesFactor* package (version 0.9.12-4.7) to compare a full model with a group effect included against the null model with no group effect. BF values interpretation considered that BF > 3 indicated moderate evidence for an effect, BF > 10 indicated strong evidence, and BF < 1/3 suggested evidence in favour of the null hypothesis. For pairwise post hoc comparisons, independent-sample Bayesian t-tests were conducted between groups using ttestBF(). Effect sizes were estimated using Bayesian credible intervals (95% highest density interval, HDI) to assess parameter uncertainty.

## 3. Results

**Figure 4:**
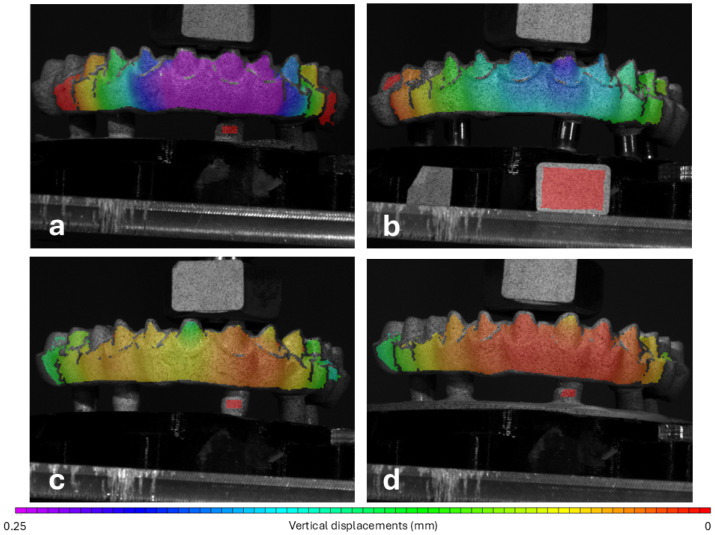
Graphical representation of the vertical displacements (V transformed axis) determined for the surface of the frameworks of (**a**) G1-PEEK, (**b**) G2-PEKK, (**c**) G3-PMMA and (**d**) G4-FRC at 200 N load. Same colour scale for all images.

All twelve polymeric frameworks resisted the load applied with no macroscopic signs of cracks or fissures. The results show that movement of the frameworks occurred generally along the direction of the applied load (vertical) and towards the cameras (anterior–posterior), with despicable lateral displacement (horizontal). The distribution of vertical displacements under 200 N load presented in Figure 4 suggests that the highest values were registered for G1-PEEK, whereas the lowest values were found in G4-FRC. The mean values of displacements under the progressive loads are detailed in Table 1. The analysis of the magnitude of displacements of the frameworks yielded a Bayes Factor of BF = 134.79 ± 0.01%, indicating very strong evidence in favour of the hypothesis of differences between groups compared to the null hypothesis of no group differences. Bayesian post hoc pairwise comparisons suggested high evidence for differences between PEEK and PEKK (BF = 39.07 ± 0.00%), PEEK and PMMA (BF = 210.39 ± 0.00%) and moderate evidence between PEEK and FRC (BF = 9.51 ± 0.00%). A weaker effect was observed between PEKK and PMMA (BF = 4.36 ± 0.00%). The comparison between PEKK and FRC (BF = 1.23 ± 0.01%) provided an inconclusive result, with no evidence for a difference between groups while the comparison between FRC and PMMA (BF = 0.58 ± 0.01%) suggested evidence in favour of the null hypothesis, indicating no meaningful difference between these groups.

As expected, both mean principal tensile and compressive surface strains increased with load increase. At 200 N, G3-PMMA presented the highest calculated mean principal tensile and compressive surface strains (Table 1).

**Figure 5:**
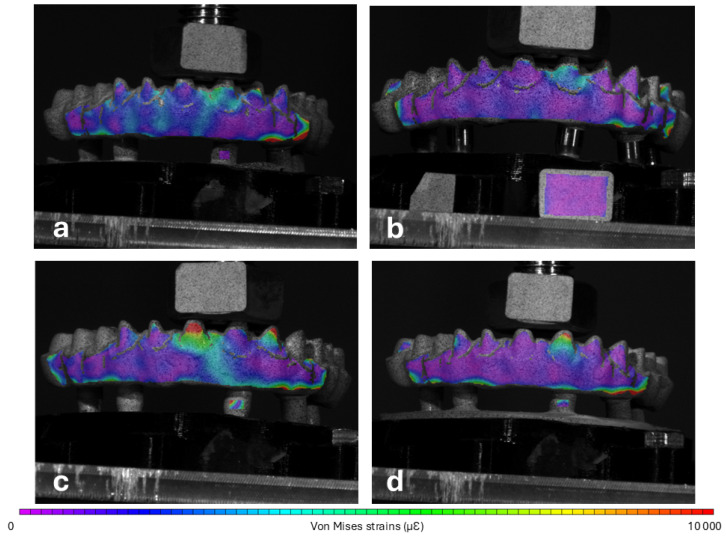
Graphical representation of the von Mises strains (ƐVM) determined for the surface of the frameworks of (**a**) G1-PEEK, (**b**) G2-PEKK, (**c**) G3-PMMA and (**d**) G4-FRC at 200 N load. Same colour scale for all images.

The von Mises strain distribution is represented in Figure 5. It is possible to notice high deformations, represented in light blue shades, crossing the G3-PMMA framework from the occlusal surface to the surface facing the implants and spreading throughout this surface. Some similar level strains are also distributed across the buccal surface of the G1-PEEK framework but with a more uniform pattern. G2-PEKK and G4-FRC present even and low-level surface strains.

At 200 N, the highest mean value of von Mises strains was found for G3-PMMA (2285.96 ± 215.17 µƐ), closely followed by G1-PEEK (2165.73 ± 105.60 µƐ). There was strong evidence of differences in mean values of von Mises strains between groups (BF = 91.00 ± 0.00%). Post hoc analysis suggested evidence in favour of the null hypothesis, indicating no meaningful difference between G1-PEEK and G3-PMMA (BF = 0.69 ± 0.00%). Strong evidence in favour of the hypothesis of a difference was found for the comparison of G4-FRC and G1-PEEK (BF = 76.88 ± 0.00%) and G3-PMMA (BF = 18.85 ± 0.00%). The remaining comparisons could not provide evidence towards the hypothesis of a difference.

## 4. Discussion

The present study evaluated the displacements and strains registered by digital image correlation on the surface of full-arch mandibular ISFPs milled from four different polymeric materials under increasing load up to 200 N. The results indicate significant differences in the magnitude of displacements and von Mises strain distribution among the tested materials, suggesting that the framework material plays a crucial role in load distribution and structural stability.

From the analysis of the patterns displayed in Figure 4, it is clear that PEEK exhibited higher vertical displacements whereas FRC and PMMA displayed the lowest values. This indicates greater flexibility of PEEK frameworks under occlusal loads and enhanced rigidity of FRC and PMMA frameworks. While these results partially reflect known mechanical properties of the materials, they also indicate that under the specific conditions, materials might behave differently. The known flexural modulus of PEEK, PEKK, PMMA and FRC, which reflects material stiffness or resistance to bending, is approximately 4.4 GPa, 3.4 GPa, 2.0 GPa and 26 GPa [22,45,46,47], respectively. The high flexural modulus of FRC could easily explain the very low displacements of those frameworks when compared to the polyether-based polymers (PEEK and PEKK), which is also in line with previous studies [20,21,45,48,49,50,51]. A summary of these findings can be found in Table 2.

The superior performance of FRC in minimising displacement and strain can be attributed to its reinforced structure, which enhances load distribution and resistance to deformation. This characteristic has been previously noted in the context of dental prosthetics, where fibre-reinforced materials exhibit enhanced durability and reduced risk of mechanical failure [31]. Conversely, the relatively high strain values in PMMA raise concerns about its long-term stability in load-bearing applications, reinforcing the necessity of cautious material selection for full-arch prosthetic frameworks.

Interestingly, the mechanical behaviour of these materials in the prosthetic framework deviates from their standard properties reported in specimen-based mechanical tests. PEEK and PEKK, despite their relatively high flexural strength in specimen-based testing (100–180 MPa), exhibited increased flexibility and displacement in the prosthetic framework, likely due to structural geometry and load distribution. Similarly, PMMA, known for its low elastic modulus (2–3 GPa), displayed higher strain values in ISFPs, which could compromise its long-term performance under functional loading [22]. In contrast, FRC, which demonstrates excellent flexural strength in standardised conditions (above 300 MPa), maintained its rigidity in the framework setting. These findings highlight the need for evaluating mechanical performance in clinically relevant conditions rather than relying solely on standardised material testing.

From a clinical perspective, these findings suggest that material selection for ISFP frameworks should balance rigidity and flexibility to optimise function, passive fit and long-term durability. FRC may provide the most stable mechanical performance among the tested materials, making it a promising candidate for full-arch implant prostheses. While PEKK also demonstrated favourable results, the increased displacement compared to FRC warrants further investigation, particularly in scenarios involving dynamic loading and long-term wear resistance. PEEK and PMMA, despite their widespread use in prosthetic applications, presented the highest strain values which might affect passive fit and long-term stability and generate potential long-term effects, including fatigue-induced microcracks, indicating potential limitations for a definitive framework.

While this study provides valuable insights into the static mechanical performance of polymeric frameworks, some limitations should be acknowledged. First, the experimental conditions were limited to static loading, which does not fully replicate the complex biomechanical environment of the oral cavity. Dynamic cyclic loading, wear and material aging play crucial roles in the long-term durability of ISFPs, and future research should investigate these factors under simulated masticatory conditions. Compliance with the standards for testing polymer-based dental materials (ISO 4049:2019 [52]) and for dynamic loading of dental implants (ISO 14801:2016 [41]) will be crucial in assessing the longevity of polymeric ISFPs.

Additionally, the influence of framework geometry on stress distribution warrants further exploration. The present study used a standardised framework design; however, variations in thickness, connector dimensions and pontic span could significantly impact mechanical performance [9,53,54,55,56]. Future studies should incorporate different framework designs and load application points to better understand their effect on stress distribution and displacement patterns. Complementary integration of computational modelling approaches, such as finite element analysis, could provide further insights into stress distribution and failure prediction in implant-supported prostheses. This would help optimise material selection, framework design and clinical protocols to enhance the long-term success of polymer-based ISFPs [57].

## 5. Conclusions

This study provides valuable evidence regarding the biomechanical behaviour of polymeric ISFP frameworks under static loading. FRC exhibited superior load-bearing characteristics, reinforcing its potential as a viable clinical alternative to metal-based ISFPs These findings contribute to the growing body of knowledge guiding material selection in implant dentistry and emphasise the need for continued research on the long-term mechanical reliability of polymer-based prosthetic frameworks.

## Figures and Tables

**Table 1:** Mean magnitude of displacements, tensile (Ɛ1), compressive (Ɛ2) and von Mises (ƐVM) strains of the frameworks under progressive load.

**Group**	**Load (N)**	**Displacement Mean ± SD (µm)**	**Ɛ1** **Mean ± SD (µƐ)**	**Ɛ2** **Mean ± SD (µƐ)**	**ƐVM** **Mean ± SD (µƐ)**
G1-PEEK	50	96.36 ± 34.72	693.55 ± 134.34	−610.07 ± 92.27	858.64 ± 53.47
	100	152.21 ± 9.26	1032.95 ± 73.67	−941.95 ± 107.08	1392.81 ± 85.82
	150	220.87 ± 3.01	1330.36 ± 25.43	−1297.15 ± 71.11	1830.72 ± 76.09
	200	257.69 ± 11.12	1580.74 ± 110.04	−1535.52 ± 174.12	2165.73 ± 105.60
G2-PEKK	50	72.92 ± 5.95	616.11 ± 75.85	−675.71 ± 294.50	850.21 ± 190.72
	100	92.95 ± 11.98	814.88 ± 25.03	−799.74 ± 295.65	1074.91 ± 206.82
	150	114.11 ± 11.06	964.35 ± 100.38	−987.93 ± 334.20	1309.28 ± 254.87
	200	134.66 ± 16.91	1088.21 ± 159.53	−1075.86 ± 274.86	1483.69 ± 260.75
G3-PMMA	50	40.95 ± 2.77	890.15 ± 134.74	−874.92 ± 131.57	1133.10 ± 62.69
	100	59.35 ± 16.39	1342.96 ± 328.18	−1181.44 ± 178.25	1622.01 ± 61.56
	150	67.00 ± 8.59	1580.66 ± 302.56	−1524.64 ± 151.81	1985.14 ± 215.66
	200	84.77 ± 11.07	1873.95 ± 501.22	−1710.32 ± 117.94	2285.96 ± 215.17
G4-FRC	50	34.22 ± 33.50	843.38 ± 191.48	−545.71 ± 84.29	830.09 ± 156.02
	100	35.36 ± 22.09	908.83 ± 48.88	−667.63 ± 64.21	949.54 ± 52.15
	150	58.66 ± 50.43	1117.07 ± 67.94	−775.20 ± 41.03	1156.69 ± 66.47
	200	74.50 ± 51.02	1205.39 ± 20.24	−896.15 ± 104.68	1267.83 ± 47.38

**Table 2:** Main differences between tested materials regarding known mechanical properties (values provided by the manufacturers or publicly available databases [22]) and observed behaviour in ISFP frameworks.

**Material**	**Flexural Strength (MPa)**	**Elastic Modulus (GPa)**	**Observed Mechanical Behaviour in ISFP Frameworks**
PEEK	165	3–4	High flexibility, increased displacement under load.
PEKK	200	5.1	Slightly stiffer than PEEK but still displays flexibility.
PMMA	50–100	2–3	High strain values, concerns over long-term performance.
FRC	540	6–15	Minimal displacement, rigidity and low strain values

## Data Availability

The original contributions presented in this study are included in the article. Further inquiries can be directed to the corresponding author.

## References

[1] Berniyanti T., Palupi R., Alkadasi B.A., Sari K.P., Putri R.I., Salma N., Prasita S., Regita A.S. (2023). Oral Health-Related Quality of Life (OHRQoL) Analysis in Partially Edentulous Patients with and without Denture Therapy. Clin. Cosmet. Investig. Dent..

[2] García-Minguillán G., Preciado A., Romeo M., Río J.D., Lynch C.D., Castillo-Oyagüe R. (2021). Differences in self-perceived OHRQoL between fully dentate subjects and edentulous patients depending on their prosthesis type, socio-demographic profile, and clinical features. J. Dent..

[3] Garg P., Klineberg I. (2022). Benefits of Contemporary Rehabilitation of Edentulism: A Statement. Int. J. Prosthodont..

[4] Feng Y., Xiao L., Fu L.L., Gosau M., Vollkommer T., Speth U., Smeets R., Rutkowski R., Friedrich R.E., Yan M. (2025). Global, Regional and National Burden of Edentulism and Periodontal Diseases from 1990 to 2021: Analysis of Risk Factors and Prediction of Trends in 2050. In Vivo.

[5] Sofi-Mahmudi A., Shamsoddin E., Khademioore S., Khazaei Y., Vahdati A., Tovani-Palone M.R. (2025). Global, regional, and national survey on burden and Quality of Care Index (QCI) of orofacial clefts: Global burden of disease systematic analysis 1990–2019. PLoS ONE.

[6] Nilsson S., Stenport V.F., Nilsson M., Göthberg C. (2022). A retrospective clinical study of fixed tooth- and implant-supported prostheses in titanium and cobalt-chromium-ceramic: 5-9-year follow-up. Clin. Oral Investig..

[7] Teigen K., Jokstad A. (2012). Dental implant suprastructures using cobalt-chromium alloy compared with gold alloy framework veneered with ceramic or acrylic resin: A retrospective cohort study up to 18 years. Clin. Oral Implants Res..

[8] Vahnström M., Johansson P.H., Svanborg P., Stenport V.F. (2022). Comparison of porcelain veneer fracture in implant-supported fixed full-arch prostheses with a framework of either titanium, cobalt-chromium, or zirconia: An in vitro study. Clin. Exp. Dent. Res..

[9] Papaspyridakos P., Sinada N., Ntovas P., Barmak A.B., Chochlidakis K. (2025). Zirconia full-arch implant prostheses: Survival, complications, and prosthetic space dimensions with 115 edentulous jaws. J. Prosthodont..

[10] Grosgogeat B., Vaicelyte A., Gauthier R., Janssen C., Le Borgne M. (2022). Toxicological Risks of the Cobalt-Chromium Alloys in Dentistry: A Systematic Review. Materials.

[11] Vaicelyte A., Janssen C., Le Borgne M., Grosgogeat B. (2020). Cobalt–Chromium Dental Alloys: Metal Exposures, Toxicological Risks, CMR Classification, and EURegulatory Framework. Crystals.

[12] Alqurashi H., Khurshid Z., Syed A.U.Y., Rashid Habib S., Rokaya D., Zafar M.S. (2021). Polyetherketoneketone (PEKK): An emerging biomaterial for oral implants and dental prostheses. J. Adv. Res..

[13] Bemelmanns P., Pfeiffer P. (2001). Shock absorption capacities of mouthguards in different types and thicknesses. Int. J. Sports Med..

[14] Menini M., Delucchi F., Bagnasco F., Baldi D., Canullo L., Setti P., Migliorati M., Simetti E., Pesce P. (2024). Shock Absorption Capacity of High-Performance Polymers for Dental Implant-Supported Restorations: In Vitro Study. Dent. J..

[15] Villefort R.F., Diamantino P.J.S., Zeidler S.L.V.V., Borges A.L.S., Silva-Concílio L.R., Saavedra G.D.F.A., Tribst J.P.M. (2022). Mechanical Response of PEKK and PEEK As Frameworks for Implant-Supported Full-Arch Fixed Dental Prosthesis: 3D Finite Element Analysis. Eur. J. Dent..

[16] Almjaddr M., Saker J. (2024). Effect of Different Cantilever Lengths in Polyether Ether Ketone Prosthetic Framework in All-on-Four Technique on Stress Distribution: A Three-Dimensional (3D) Finite Element Analysis. Cureus.

[17] Elsayed S., Ahmed Y., El-Anwar M.I., Elddamony E., Ashraf R. (2025). Influence of different polymeric materials of implant and attachment on stress distribution in implant-supported overdentures: A three-dimensional finite element study. BMC Oral Health.

[18] Kilic S., Caglar I. (2024). An Investigation of Stress Distribution Between Two Different Implant Concept in Implant-Supported Maxillary Prostheses with Different Framework Materials: A Finite Element Study. Int. J. Prosthodont..

[19] Ogawa T., Dhaliwal S., Naert I., Mine A., Kronstrom M., Sasaki K., Duyck J. (2010). Impact of implant number, distribution and prosthesis material on loading on implants supporting fixed prostheses. J. Oral Rehabil..

[20] Reddy K.U.K., Seth A., Vuppuluri A., Verma P.C., Narala S.K.R., Babu P.J., Saravanan P. (2025). Exploring the bio-mechanical behavior of PEEK and CFR-PEEK materials for dental implant applications using finite element analysis. J. Prosthodont. Res..

[21] Sahin Hazir D., Sozen Yanik I., Guncu M.B., Canay R.S. (2025). Biomechanical behavior of titanium, cobalt-chromium, zirconia, and PEEK frameworks in implant-supported prostheses: A dynamic finite element analysis. BMC Oral Health.

[22] LLC M.t. MatWeb: Online Materials Information Resource. http://www.matweb.com.

[23] Dinçtürk B A., Garoushi S., Alp C K., Pk V., Mb Ü., Lassila L. (2025). Fracture resistance of endocrowns made from different CAD/CAM materials after prolonged fatigue aging. Clin. Oral Investig..

[24] Breitman L.S., Alsahafi T., Kofford B., Felton D.A., Prasad S. (2025). Flexural strength and mode of failure of interim implant-supported fixed dental prostheses following different conversion techniques and structural reinforcement. J. Prosthet. Dent..

[25] Bijelic-Donova J., Bath A.K., Rocca G.T., Bella E.D., Saratti C.M. (2025). Can Fiber-reinforced Composites Increase the Fracture Resistance of Direct Composite Restorations in Structurally Compromised Teeth? A Systematic Review and Meta-analysis of Laboratory Studies. Oper. Dent..

[26] Lahoud L., Boulos P., Kahale D., Gheno E., Benedicenti S., Calasans-Maia M.D., Bassano M.B., Signore A., Dawalibi A., Nasr E. (2024). Fracture load comparison of a new Fiber-Reinforced Composite and Zirconia in All-on-Four Prosthesis: An In Vitro Study. Int. J. Prosthodont..

[27] Garoushi S., Barlas D., Vallittu P.K., Uctasli M.B., Lassila L. (2024). Fracture behavior of short fiber-reinforced CAD/CAM inlay restorations after cyclic fatigue aging. Odontology.

[28] Corbani K., Hardan L., Eid R., Skienhe H., Alharbi N., Ozcan M., Salameh Z. (2021). Fracture Resistance of Three-unit Fixed Dental Prostheses Fabricated with Milled and 3D Printed Composite-based Materials. J. Contemp. Dent. Pract..

[29] Cahyanto A., Martins M.V.S., Bianchi O., Sudhakaran D.P., Sililkas N., Echeverrigaray S.G., Rosa V. (2023). Graphene oxide increases PMMA’s resistance to fatigue and strength degradation. Dent. Mater..

[30] Facenda J.C., Borba M., Benetti P., Borges A.L.S., Dutra M.D.Z., Corazza P.H. (2023). Fatigue resistance of polymeric restorative materials: Effect of supporting substrate. Gen. Dent..

[31] Magne P., Milani T. (2023). Short-fiber Reinforced MOD Restorations of Molars with Severely Undermined Cusps. J. Adhes. Dent..

[32] Alarcon J.V., Engelmeier R.L., Powers J.M., Triolo P.T. (2009). Wear testing of composite, gold, porcelain, and enamel opposing a removable cobalt-chromium partial denture alloy. J. Prosthodont..

[33] Domagała I., Przystupa K., Firlej M., Pieniak D., Gil L., Borucka A., Naworol I., Biedziak B., Levkiv M. (2021). Analysis of the Statistical Comparability of the Hardness and Wear of Polymeric Materials for Orthodontic Applications. Materials.

[34] Harrison A., Huggett R., Handley R.W. (1979). A correlation between abrasion resistance and other properties of some acrylic resins used in dentistry. J. Biomed. Mater. Res..

[35] Jain S., Sayed M.E., Shetty M., Alqahtani S.M., Al Wadei M.H.D., Gupta S.G., Othman A.A.A., Alshehri A.H., Alqarni H., Mobarki A.H. (2022). Physical and Mechanical Properties of 3D-Printed Provisional Crowns and Fixed Dental Prosthesis Resins Compared to CAD/CAM Milled and Conventional Provisional Resins: A Systematic Review and Meta-Analysis. Polymers.

[36] Mackert J., El-Shewy M., Pannu D., Schoenbaum T. (2024). Prosthetic complications and survival rates of metal-acrylic implant fixed complete dental prostheses: A retrospective study up to 10 years. J. Prosthet. Dent..

[37] Palaniappan S., Celis J.P., Van Meerbeek B., Peumans M., Lambrechts P. (2013). Correlating in vitro scratch test with in vivo contact free occlusal area wear of contemporary dental composites. Dent. Mater..

[38] Almuhayya S., Alshahrani R., Alsania R., Albassam A., Alnemari H., Babaier R. (2025). Biofilm Formation on Three High-Performance Polymeric CAD/CAM Composites: An In Vitro Study. Polymers.

[39] Khoury P., Kharouf N., Etienne O., Dillenseger J.P., Haikel Y., El-Damanhoury H.M., Irani D., Ozcan M., Salameh Z. (2024). Physicochemical Properties and Bacterial Adhesion of Conventional and 3D Printed Complete Denture PMMA Materials: An. J. Contemp. Dent. Pract..

[40] Shamieh S., Ribeiro A.A., Sulaiman T., Swift E.J., Vasconcellos A.B. (2024). Biofilm attachment and mineralizing potential of contemporary restorative materials. Am. J. Dent..

[41] (2016). Dentistry—Implants—Dynamic Loading Test for Endosseous Dental Implants.

[42] Schimmel M., Araujo M., Abou-Ayash S., Buser R., Ebenezer S., Fonseca M., Heitz-Mayfield L.J., Holtzman L.P., Kamnoedboon P., Levine R. (2023). Group 4 ITI Consensus Report: Patient benefits following implant treatment in partially and fully edentulous patients. Clin. Oral Implants Res..

[43] Srinivasan M., Kamnoedboon P., Angst L., Müller F. (2023). Oral function in completely edentulous patients rehabilitated with implant-supported dental prostheses: A systematic review and meta-analysis. Clin. Oral Implants Res..

[44] Messias A., Karasan D., Nicolau P., Pjetursson B.E., Guerra F. (2023). Rehabilitation of full-arch edentulism with fixed or removable dentures retained by root-form dental implants: A systematic review of outcomes and outcome measures used in clinical research in the last 10 years. J. Clin. Periodontol..

[45] Schwitalla A.D., Spintig T., Kallage I., Müller W.D. (2015). Flexural behavior of PEEK materials for dental application. Dent. Mater..

[46] Yerliyurt K., Taşdelen T.B., Eğri Ö., Eğri S. (2023). Flexural Properties of Heat-Polymerized PMMA Denture Base Resins Reinforced with Fibers with Different Characteristics. Polymers.

[47] Bioloren TRILOR®: The Solution for a Metal Free Dentistry. https://bioloren.com/english/trilor-fiber-disks-and-blocks.

[48] Tushar, Rani P., Ananya, Kumar S., Prakash J., Jayaprakash M.B. (2023). Evaluation of Impact Strength and Flexural Strength of Polyether Ether Ketone vs. Computer-Aided Design/Computer-Aided Manufacturing Polymethyl Methacrylate Denture Base Materials: An In-Vitro Study. Cureus.

[49] Valenti C., Federici M.I., Coniglio M., Betti P., Pancrazi G.P., Tulli O., Masciotti F., Nanussi A., Pagano S. (2024). Mechanical and biological properties of polymer materials for oral appliances produced with additive 3D printing and subtractive CAD-CAM techniques compared to conventional methods: A systematic review and meta-analysis. Clin. Oral Investig..

[50] Alghazzawi T.F. (2023). Relation of Crown Failure Load to Flexural Strength for Three Contemporary Dental Polymers. Polymers.

[51] Ruschel G.H., Gomes É., Silva-Sousa Y.T., Pinelli R.G.P., Sousa-Neto M.D., Pereira G.K.R., Spazzin A.O. (2018). Mechanical properties and superficial characterization of a milled CAD-CAM glass fiber post. J. Mech. Behav. Biomed. Mater..

[52] (2019). Dentistry—Polymer-Based Restorative Materials.

[53] Muhsin S.A., Mohammed E.K., Bander K. (2024). Finite Element Analysis: Connector Designs and Pontic Stress Distribution of Fixed Partial Denture Implant-Supported Metal Framework. J. Long Term Eff. Med. Implants.

[54] Huang L.S., Huang Y.C., Yuan C., Ding S.J., Yan M. (2023). Biomechanical evaluation of bridge span with three implant abutment designs and two connectors for tooth-implant supported prosthesis: A finite element analysis. J. Dent. Sci..

[55] Luft R.L., da Rosa L.S., Machado P.S., Valandro L.F., Sarkis-Onofre R., Pereira G.K.R., Bacchi A. (2022). Influence of connector cross-sectional geometry on the load-bearing capacity under fatigue of implant-supported zirconia fixed partial prosthesis. J. Prosthet. Dent..

[56] Alshiddi I.F., Habib S.R., Zafar M.S., Bajunaid S., Labban N., Alsarhan M. (2021). Fracture Load of CAD/CAM Fabricated Cantilever Implant-Supported Zirconia Framework: An In Vitro Study. Molecules.

[57] Pjetursson B.E., Fehmer V., Sailer I. (2022). EAO Position Paper: Material Selection for Implant-Supported Restorations. Int. J. Prosthodont..

